# Endoscopic intermuscular dissection combined with an absorbable string-loop suture for salvaging a residual rectal lesion with severe fibrosis after endoscopic submucosal dissection: A case description

**DOI:** 10.1055/a-2878-1177

**Published:** 2026-06-09

**Authors:** Shibo Song, Yunlong Cai, Yajie Wang, Xiaolong Rao, Long Rong

**Affiliations:** 1 Endoscopy Center 26447 Peking University First HospitalBeijingBeijingChina

Residual rectal lesions after endoscopic submucosal dissection (ESD) pose a dilemma: radical surgery risks a permanent stoma, while repeat ESD is hindered by extensive submucosal fibrosis. Recent studies have demonstrated that endoscopic intermuscular dissection (EID) offers a viable rescue alternative with high R0 resection rates. 1​ 2​ ​3​ To the best of our knowledge, we report the first case of EID combined with an absorbable string-loop suture (ALS) for a suspected residual low rectal lesion following non-curative ESD.

A 52-year-old man was referred to our center 1 month after a non-curative ESD for a 2-cm rectal laterally spreading tumor, pathologically confirmed as a well-differentiated adenocarcinoma (pT1) with a close vertical margin. Colonoscopy revealed significant scarring and residual clips ( Fig. 1 ). Following multidisciplinary evaluation, EID and ALS were performed ( Video 1 ). After circumferential incision and initial submucosal dissection, the circular muscularis propria was incised to access the intermuscular space ( Fig. 2 ). Due to severe fibrosis and muscle retraction, a portion of the longitudinal muscularis propria was also resected ( Fig. 3 ). The 4-cm defect, located 2 cm from the anal verge, presented a significant technical challenge for closure. Using the ALS technique previously described by our team, the string-loop was anchored to the midpoints of the defect edges with clips. 4​ Tightening the sliding knot reshaped the large oval defect into two smaller ones, facilitating complete closure with additional clips ( Figs. 4 and 5 ). Resection and closure took 48 and 16 minutes, respectively. Postoperative day 3 endoscopy confirmed stable closure. Pathology revealed no residual tumor, and the patient was discharged uneventfully on day 4.

**Fig. 1:**
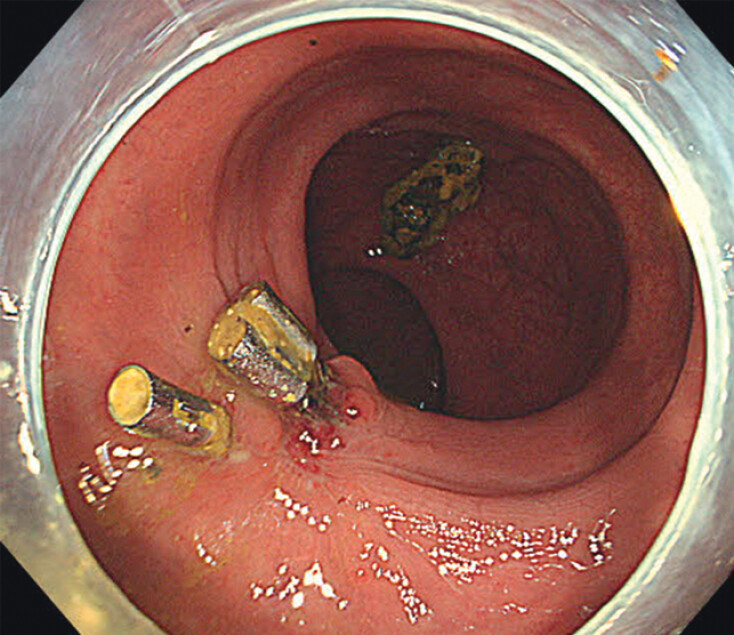
Follow-up colonoscopy at 1 month reveals significant scarring and residual clips at the previous ESD site. ESD, endoscopic submucosal dissection.

**Video 1** Successful rescue of a post-ESD residual rectal lesion with severe fibrosis using endoscopic intermuscular dissection combined with an absorbable string-loop suture. ESD, endoscopic submucosal dissection.


**Fig. 2:**
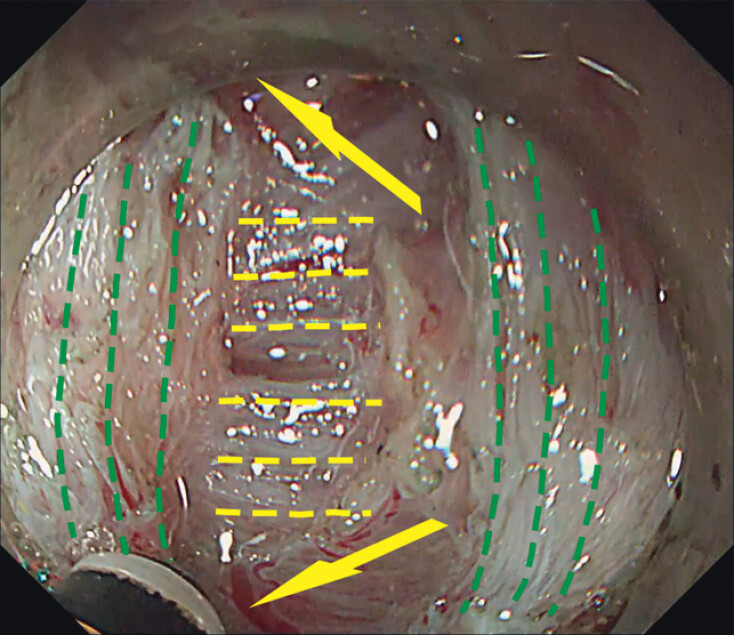
Dissection along the intermuscular space. Notes: Green dashed lines indicate the circular muscle layer, yellow dashed lines indicate the longitudinal muscle layer, and yellow arrows show the direction of dissection.

**Fig. 3:**
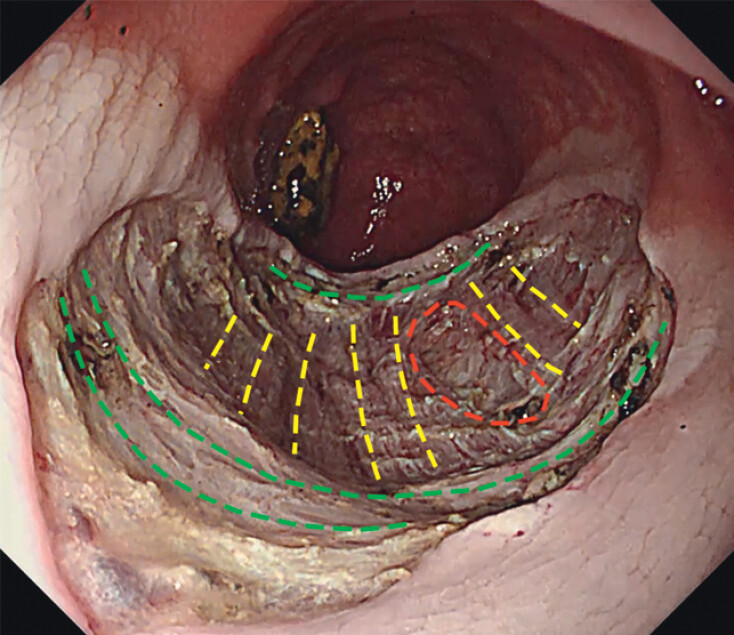
The post-endoscopic intermuscular dissection defect with partial resection of the longitudinal muscularis propria. Notes: Green dashed lines indicate the circular muscle layer, yellow dashed lines indicate the longitudinal muscle layer, and red circles highlight the exposed mesorectum.

**Fig. 4:**
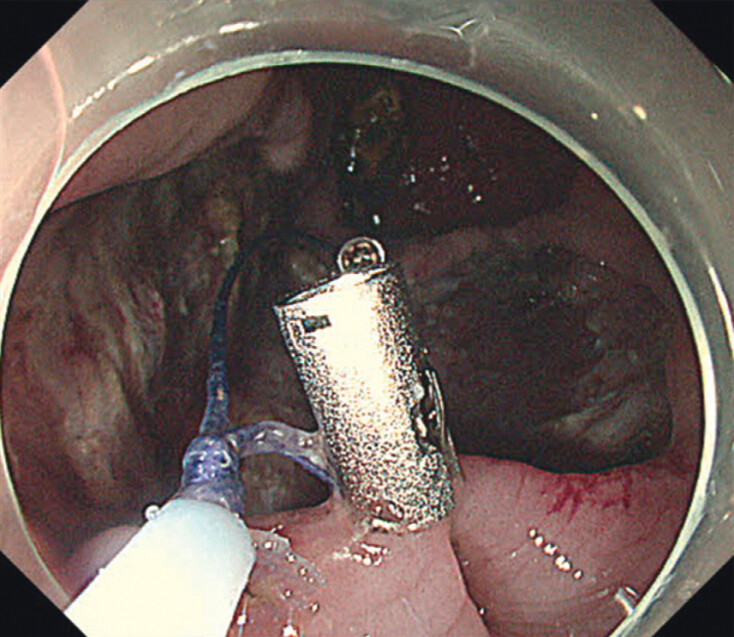
The sliding knot on the absorbable string-loop is tightened via a nested sheath.

**Fig. 5:**
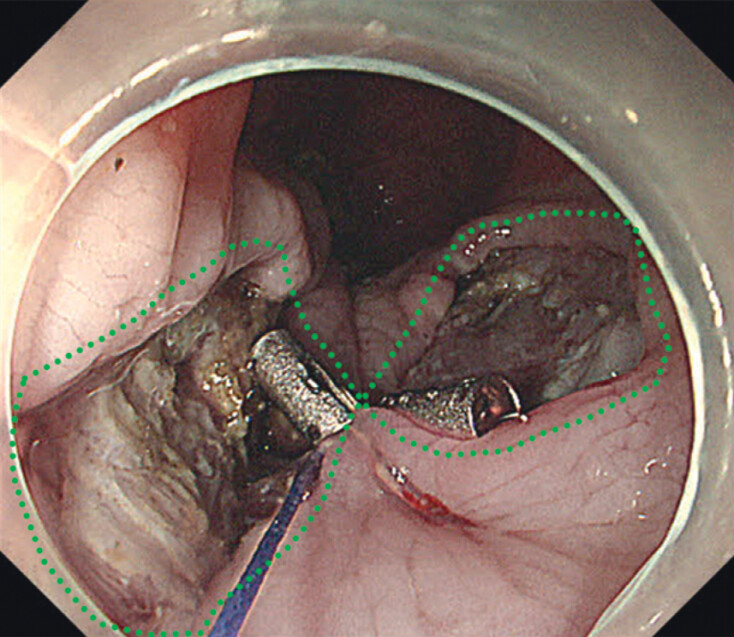
The large oval defect is reshaped into two smaller ones. Note: Green circles indicate the extent of the defect.

EID combined with ALS successfully salvaged the non-curative rectal lesion, sparing the patient from radical surgery and effectively closing the deep defect. This combined strategy offers a viable alternative for challenging rectal lesions with severe fibrosis. Further clinical validation is warranted.

Endoscopy_UCTN_Code_TTT_1AQ_2AK

## Informed Consent

Written informed consent was obtained from the patient for the surgery and for the publication of this case report and any accompanying images.
